# Current Status of Cooperation Between Obstetric Care Institutes and Psychiatrists in Japan

**DOI:** 10.7759/cureus.71413

**Published:** 2024-10-14

**Authors:** Yoko Sagara, Shunji Suzuki, Shin-ichi Hoshi, Akihiko Sekizawa, Isamu Ishiwata

**Affiliations:** 1 Division of Maternal and Child Health, Japan Association of Obstetricians and Gynecologists, Tokyo, JPN; 2 Department of Obstetrics and Gynecology, Tokyo Riverside Hospital, Tokyo, JPN; 3 Department of Obstetrics and Gynecology, Showa University, Tokyo, JPN

**Keywords:** cooperation, japan, obstetric care institutes, perinatal mental health, psychiatrists

## Abstract

In May 2024, we requested all obstetric institutes (n = 1970) that are members of the Japan Association of Obstetricians and Gynecologists (JAOG) to provide information concerning the collaboration between obstetric institutes and psychiatrists in Japan, such as (1) pregnancy and delivery management of all pregnant women with psychiatric complications, and (2) availability of smooth collaboration with associated psychiatry (or psychiatrists) regarding pregnant women with psychiatric complications. Of the 1970 obstetric institutes, 1483 (75.2%) responded with valid information (completion of data submitted). In Japan, only 21% (308/1483) of all obstetric institutes are able to see all pregnant women with psychiatric complications who attend their hospital, and 32% (480/1483) of the institutes responded that they have smooth cooperation with associated psychiatrists. The response to pregnant women with mental health problems in Japanese obstetric institutes is not adequately equipped yet.

## Introduction

According to the interviews with municipalities conducted in recent years in Japan, a system of information-sharing and cooperation between obstetric institutes and municipalities has mostly been built, and pediatric care institutes can also provide support according to the growth and development of the child [1-3]. In addition, various guidelines have been published for various occupations according to the level of perinatal mental problems that need to be addressed [4-6]. However, unfortunately, there are few psychiatric institutes where mothers raising infants can be examined, and cooperation between obstetric institutes and psychiatry is observed to be still insufficient in Japan [7,8].

In Japan, there are three systems of obstetric institutes: private clinics, general hospitals without neonatal intensive care units (NICU), and perinatal centers (hospitals with permanent NICUs) [9,10]. Obstetric care at private clinics has tended to involve good and comfortable communication between the obstetricians and the pregnant women; however, it will not be able to handle high-risk pregnancies [11-13]. In private clinics, pregnancies and deliveries are managed by one or two full-time obstetricians along with several midwives. According to data from the Japan Ministry of Health, Labor and Welfare, the percentage of hospitals or perinatal centers with a psychiatrist on staff is approximately 20%. Therefore, in private clinics and about 80% of hospitals in Japan, pregnant women who need to see a psychiatrist have been referred to psychiatrists at another institute [8-10]. Because pregnant and postpartum women suffering from mental disorders need to be treated while considering the effects on the fetuses and breastfeeding, psychiatrists are, unfortunately, sometimes reluctant to care for them in Japan, and referrals can be difficult [14-16]. In Japan, half of all deliveries are managed in obstetric clinics run by individuals; therefore, such institutes may have difficulty approaching psychiatry when necessary [17].

In order to ascertain such a situation in Japan, we conducted a questionnaire survey of all obstetric institutes throughout Japan regarding their cooperation with psychiatrists.

## Materials and methods

The protocol for this study was approved by the Ethics Committee of the Japan Association of Obstetricians and Gynecologists (JAOG) (No. 202403).

In May 2024, we requested the heads of the obstetrics department at all obstetric institutes (n = 1970) that are members of the JAOG to provide information concerning (1) maternal mental health screening for all women such as a history of mental disorders, and/or current depression, and/or anxiety during pregnancy, (2) maternal mental health screening of current depression and/or anxiety for all women at one month after delivery (as postpartum), (3) pregnancy and delivery management of all pregnant women with psychiatric complications, (4) availability of smooth collaboration with associated (affiliated) psychiatry (or psychiatrists), and (5) availability of smooth cooperation with local administrative agencies regarding pregnant women with psychiatric complications. The questions were worded as simply as possible to improve the response rate, and the answers were collected as yes or no. The questions were prepared by modifying the questionnaire used in our previous report, which suggested that the number of obstetric institutes managing psychiatric disorders was inadequate in Japan [7]. The questions were mailed to each obstetric institute, and responses were received via fax or an Internet survey form.

During the study period, the number of private clinics, general hospitals without a NICU, and perinatal centers were 1042 (53%), 527 (27%), and 401 (20%), respectively.

In this study, based on the answers obtained for the above (1) through (5), we analyzed the actual status of efforts toward perinatal mental health care with collaboration between obstetrics and psychiatry in obstetric institutes in Japan as a whole and by the obstetric practice system.

Data are presented as numbers and percentages (%). The statistical software SAS version 8.02 (SAS Institute, Cary, NC, USA) was used for statistical analyses. A chi-square test and Fisher's exact test were used for statistical analyses, and a p-value of < 0.05 was considered significant.

## Results

In this study, 1483 of 1970 obstetric institutes (75.2%) responded with valid information (the completion of data submitted). The response rate of the obstetric institutes by practice system was 74.0% (771/1042) for private clinics, 73.6% (388/527) for general hospitals, and 78.3% (314/401) for perinatal centers, respectively, and there were no significant differences in the response rates by obstetric practice system.

Table 1 shows the management of pregnant women with mental health problems in Japanese obstetric institutes by each practice system. As shown in Table 1, in Japan, perinatal centers had the highest implementation rates for the screening of maternal mental status, management of pregnant women with psychiatric complications, and collaboration with associated psychiatry and local government agencies while private obstetric clinics had the lowest implementation rates for them. In Japan as a whole, only 21% (308/1483) of all obstetric institutes are able to see all pregnant women with psychiatric complications who attend their hospital, and even when restricted to perinatal centers alone, 53% (165/314) of all centers are able to see all pregnant women with psychiatric complications who attend their hospital. On the other hand, 32% (480/1483) of all institutes responded that they have smooth cooperation with associated psychiatrists. Thirty-six percent (36%; 480-308/480) of the obstetric institutes did not see pregnant women with psychiatric complications, even though they had associated psychiatrists.

**Table 1:** Management of pregnant women with mental health problems in Japanese obstetric institutes by each practice system Data are presented as numbers (percentages). NICU, neonatal intensive care unit *< 0.05 vs. private clinics; # < 0.05 vs. general hospitals without NICU

	Private clinic	General hospitals without NICU	Perinatal center	Total
Total	771	398	314	1483
Maternal mental health screening during pregnancy	167 (22)	110 (28)*	173 (55)*#	450 (30)
Maternal mental health screening at postpartum	296 (38)	162 (41)	233 (74)*#	691 (47)
Management of pregnant women with psychiatric complications	61 (8)	82 (21)*	165 (53)*#	308 (21)
Smooth collaboration with associated psychiatry (or psychiatrists)	115 (15)	143 (36)*	222 (71)*#	480 (32)
In-house psychiatrist	9 (1)	125 (31)*	220 (70)*#	354 (24)
Smooth cooperation with local administrative agencies	180 (23)	116 (29)	139 (44)*#	435 (29)

On the other hand, 35% (271/771), 56 (219/388), and 42% (131/314) of the private clinics, general hospitals, and perinatal centers, respectively, indicated that they work in collaboration with the family psychiatrists of the pregnant women with psychiatric complications.

## Discussion

Although the importance of perinatal mental health care has been well-recognized in Japan [10,15,17,18], unfortunately, it has become clear that the response to pregnant women with mental health problems in Japanese obstetric institutes is not yet adequately equipped. Even if they were perinatal centers, half of the institutes did not appear to be able to manage all pregnant women with psychiatric disorders. In other words, more than half of the obstetric institutes in any of the practice systems have appeared to be dealing with pregnant women with psychiatric complications in collaboration with an unspecified psychiatrist at another institute. Although this collaboration has been compensated for in the Japanese insurance system, we cannot deny the possibility that psychotherapy may be ineffective. In addition, it became clear once again that many obstetric institutes in Japan are unable to manage pregnant women with complications of serious mental disorders, not only because there are no mother-baby units in psychiatric wards but also because there are psychiatrists but no psychiatric wards. For example, a pregnant woman with psychiatric disorders requiring hospitalization may be transported to a medical facility far from home. Japan may not be a large country; however, this will put additional stress on pregnant women with mental health problems. In other words, it is presumed that Japan needs to secure psychiatric institutes for perinatal mental health beyond technology-based interventions for perinatal mental health [19].

Obstetric care at private clinics has tended to involve good and comfortable communication between obstetricians and pregnant women; however, it will not be able to handle high-risk pregnancies [11-13]. Although the staff at obstetric private clinics might have been likely trying their best, unfortunately, the feared results have emerged concerning perinatal mental health care.

In Japan, there are 720,000 deliveries per year, managed by approximately 2000 obstetric institutes and 12000 obstetricians and gynecologists [9]. On the other hand, in Japan, it is reported that there are about 10,000 psychiatrists and approximately 38000 midwives. While Japan has the advantage of having a large number of medical facilities, including obstetric institutes, and most citizens can receive most medical care close to home, the disadvantage is that there is an insufficient concentration of medical specialists, making it difficult for patients with multiple complications to find adequate medical facilities to be seen. The current results may indicate some of these Japanese medical characteristics. Although the appropriate number of physicians and midwives is unknown, the dispersion among many institutes may lead to a narrowing of the scope of practice at individual institutes. Therefore, in Japan, it may be necessary to reform the regional medical system with consolidation or to establish a mandatory system of cooperation between obstetrics and psychiatry by the central government. We also need to learn how countries with fewer physicians than Japan have solved the problem of ‘screening and early intervention’ by non-psychiatrists although there is a large difference in the background of pregnant women [20].

We understand that there are some limitations in this study. First, the current surveys had 75% response rates; however, the obstetric institutes that responded may be more interested in perinatal mental problems than those that did not respond. Thus, the current data may not accurately represent the reality of the medical management of pregnancies with psychiatric problems in Japan. Second, in this study, the questions were worded as simply as possible to improve the response rate. Lack of collaboration between obstetric care providers and psychiatrists can lead to delays in responding to women with mental health problems, which can have dire consequences [21]. They will contribute to the status of collaboration as well as the existence of collaboration. Therefore, while this study examined the availability of dealing with pregnancies with mental disorders and affiliated psychiatrists, the level of management or effectiveness was not examined. In addition, since the current survey was conducted among obstetricians who are members of the JAOG, it is unclear whether accurate responses regarding effective medical coordination systems were obtained. Therefore, it is presumed that additional surveys on perinatal outcomes consideration of interventional methods will be needed to accurately assess the effectiveness of the perinatal psychiatric coordination system in Japan.

## Conclusions

According to the interviews with obstetric institutes in Japan, only 21% of all obstetric institutes were able to see all pregnant women with psychiatric complications who attended their hospital, and 32% of the institutes responded that they had smooth cooperation with associated psychiatrists. The response to pregnant women with mental health problems in Japanese obstetric institutes is not adequately equipped yet.
